# Blood type and breed-associated differences in cell marker expression on equine bone marrow-derived mesenchymal stem cells including major histocompatibility complex class II antigen expression

**DOI:** 10.1371/journal.pone.0225161

**Published:** 2019-11-20

**Authors:** J. Lacy Kamm, Natalie A. Parlane, Christopher B. Riley, Erica K. Gee, Keren E. Dittmer, C. Wayne McIlwraith

**Affiliations:** 1 Massey University, School of Veterinary Science, Massey University, Palmerston North, New Zealand; 2 Veterinary Associates, Karaka, Auckland, New Zealand; 3 AgResearch, Hopkirk Research Institute, Massey University, Palmerston North, New Zealand; 4 Colorado State University, Orthopaedic Research Center, Fort Collins, Colorado, United States of America; MERLN Institute, Maastricht University, NETHERLANDS

## Abstract

**Background:**

As the search for an immune privileged allogeneic donor mesenchymal stem cell (MSC) line continues in equine medicine, the characterization of the cells between different sources becomes important. Our research seeks to more clearly define the MSC marker expression of different equine MSC donors.

**Methods:**

The bone marrow-derived MSCs from two equine breeds and different blood donor-types were compared over successive culture passages to determine the differential expression of important antigens. Eighteen Thoroughbreds and 18 Standardbreds, including 8 blood donor (erythrocyte Aa, Ca, and Qa antigen negative) horses, were evaluated. Bone marrow was taken from each horse for isolation and culture of MSCs. Samples from passages 2, 4, 6, and 8 were labelled and evaluated by flow cytometry. The cell surface expression of CD11a/18, CD44, CD90 and MHC class II antigens were assessed. Trilineage assays for differentiation into adipogenic, chondrogenic and osteogenic lines were performed to verify characterization of the cells as MSCs.

**Findings:**

There were significant differences in mesenchymal stem cell marker expression between breeds and blood antigen-type groups over time. Standardbred horses showed a significantly lower expression of MHC class II than did Thoroughbred horses at passages 2, 4 and 6. CD90 was significantly higher in universal blood donor Standardbreds as compared to non-blood donor Standardbreds over all time points. All MSC samples showed high expression of CD44 and low expression of CD11a/18.

**Conclusions:**

Universal blood donor- type Standardbred MSCs from passages 2–4 show the most ideal antigen expression pattern of the horses and passages that we characterized for use as a single treatment of donor bone marrow-derived MSCs. Further work is needed to determine the significance of this differential expression along with the effect of the expression of MHC I on equine bone marrow-derived MSCs.

## Introduction

Selecting the optimal stem cell source is critical for obtaining favorable results from their use in regenerative medicine [1]. This has led to an ongoing search for mesenchymal stem cells (MSCs) with the best capacity to replace or restore function to damaged tissues and a low occurrence of side effects [2]. In equine medicine, autologous MSCs derived from bone marrow are frequently used in research and clinical cases as their ability to enhance repair of tissues damaged by musculoskeletal disease is supported by a growing body of evidence from experimental and clinical studies [3–5].

There is a move in equine medicine to use allogeneic MSCs instead of autologous MSCs due in part to the immediate availability of allogeneic MSCs and the inconsistent quality of autologous cells [6–9]. Perhaps the most important advantage of an allogeneic source of MSCs is the benefit afforded by a uniform MSC treatment for efficacy research into the therapeutic use of MSCs for equine diseases. An allogeneic cell line with a consistent phenotype would allow patients in clinical trials to be treated with MSCs from the same donor, and therefore all cases would receive a repeatable treatment. The current use of autologous MSCs in clinical studies adds an element of variability in the therapeutic efficacy of MSCs and standardized comparisons in clinical trials [10]. MSC function has been shown to vary in older humans, and the cell phenotype can vary from one bone marrow draw to the next [7– 9].

When considering treatment with allogeneic MSCs, the potential for immunologic reactions by the host is a likely cause of treatment failure [2, 7, 11]. MSCs are acutely or progressively rejected by the cell-mediated and humoral arms of the immune system leading to MSC death and local inflammation [12–14]. The major histocompatibility complex (MHC) class I and II molecules present on the cell surface facilitate allorecognition when foreign cells are transplanted into a recipient [9, 11, 15]. MHC class I and II molecules on the surface of the donor MSCs are identified by the recipient’s immune system leading to T and B lymphocyte activation [9, 11].

In horses, MHC class I molecules are expressed by most cells of the body including equine bone marrow-derived MSCs [9, 16]. The appearance of MHC class I on the cell surface causes immunorecognition and antibody formation when administered in an allogeneic manner [8, 12]. This reaction becomes apparent on serologic testing no less than seven days after administration of the foreign MSCs [8, 12]. This allorecognition may be eliminated or reduced by matching of the donor and recipient, to administer cells with MHC antigens that are as similar as possible to that of the donor [12, 15, 17]. The need for donor-recipient genotype matching (haplotyping) is currently under investigation, as some studies have shown no significant immune response to one injection of MHC I-nonmatched allogeneic MSC administration *in vivo* [2, 18, 19]. Additionally, a beneficial therapeutic effect has been seen with the use of one injection of MHC I-nonmatched allogeneic MSCs *in vivo* [18, 19].

Unlike MHC class I expression, MHC class II expression on equine bone marrow-derived MSCs varies from almost non-existent to high from one horse to another [9, 16, 20]. MHC class II expression by equine MSCs may predispose these cells to immune recognition when used in an allogeneic manner [9]. MHC class II is known to activate the innate immune system which causes a rapid immune response and T lymphocyte proliferation [9]. In horses, those MSCs expressing MHC class I and not MHC class II have been shown to not cause T cell proliferation [9]. This leads one to believe that MHC class II is possibly the primary antigen for acute cell mediated allorecognition in the horse, while both MHC class I and II cause an adaptive immune response driven by alloantibodies [8, 9, 12, 17].

Several cell surface markers are important for MSC identification and exclusion of non-MSCs. CD44 and CD90 are consistently considered as markers for MSC identification [20–24]. MSC markers CD44 and CD90 are used as inclusion markers to confirm the identity of the cells as MSCs. CD11a/18 is used in our study to show contaminating cells and is commonly an exclusion marker for MSCs in culture [23, 25, 26].

We hypothesize that one group of equids of a particular phenotype may have differing antigen expression on their MSCs as compared to another group of equids. Previous research has demonstrated that erythrocyte and leukocyte antigen expression varies between horse breeds [27, 28]. Furthermore, it is well known that a series of erythrocyte antigens causes immune reaction leading to hemolysis after blood transfusion [29]. Hematopoietic stem cells and MSCs have common lineage at the embryonic level, though literature has shown that their relationship may continue to adulthood [30]. We intend to determine if there is some correlation between the expression of immunogenic antigens on erythrocytes and those immunogenic antigens that are expressed on MSCs. For this reason MSC marker expression from cells sourced from universal blood donor type horses and non-blood donor type horses were compared. The effect of blood donor status on MSC phenotype has not previously been described in horses.

The aim of this study was to determine the frequency of expression of several cell markers in populations of MSCs derived from Thoroughbreds, Standardbreds and horses characterized as universal blood donor horses. Thoroughbreds and Standardbreds were chosen for comparison as they are two common breeds in New Zealand and many other countries, and these horses suffer from injuries that may benefit from treatment with MSCs [4, 31, 32]. Additionally, these breeds are known to have differences in erythrocyte antigen expression as a Standardbred horse is more likely to be a universal blood donor as compared to a Thoroughbred [27]. The study sought to determine if one a particular phenotype of equids studied has an MSC passage number that yielded bone marrow-derived MSCs with the most ideal cell surface antigen presentation that would decrease recipient immune system recognition (low MHC II expression) while showing optimal ability to proliferate and differentiate (high CD44 and CD90 expression).

## Materials and methods

### Experimental design

In brief, 36 horses were classified into groups according to their breed and erythrocyte antigen status. These included registered Thoroughbreds (n = 18) and Standardbreds (n = 18) of ages ranging from 2–13 years (median 4 years, interquartile range 4–6 years). Of the Standardbreds, 8 were erythrocyte antigen negative (blood donor type) and 10 were positive for erythrocyte antigens (non-blood donors). None of the Thoroughbreds were erythrocyte antigen negative. All of the horses were either owned independently or by Massey University and consent for their use was granted by all parties. Bone marrow was harvested from horses for MSC culture. MSC samples taken from passage 2, 4, 6 and 8 were assessed for their surface marker phenotype using flow cytometry. Trilineage testing was performed on a sample from each group of horses.

### Bone marrow harvest, isolation and culture

Following ethics approval by the Massey University Animal Ethics Committee (MUAEC Protocol 15/13), MSCs were harvested from the sternum of all 36 horses. In brief, 15 mL of bone marrow was aseptically harvested and added to 3 mL of 1000 IU/mL heparin (Pfizer®, New York, NY, USA), using previously described techniques [33]. Blood (25 mL) was collected via the jugular vein and placed in blood tubes (Rapid Serum Tube, BD Vacutainer®, San Jose, CA, USA) for serum collection. The bone marrow aspirates and blood tubes were transported to the laboratory on cold saline bags (3–5°C).

MSCs were isolated within 12 hours of harvest. Bone marrow aspirates were centrifuged at 200 X *g* at room temperature for 2 minutes. The supernatant was centrifuged at 1,000 X *g* for 10 minutes to pellet the nucleated cells. The supernatant was discarded and the pellet suspended in low-glucose Dulbecco modified Eagle’s medium (DMEM, Gibco^TM^, Thermo Fisher®, Waltham, MA, USA) with 10% fetal bovine serum (FBS, Gibco^TM^, Thermo Fisher®), penicillin (100 IU/ml), streptomycin (100 ug/ml) and amphotericin B (0.25ug/ml) (Sigma-Aldrich®, St Louis, MO, USA) and 2.5% 1M HEPES buffer (Gibco^TM^, Thermo Fisher®). The same FBS batch was used throughout the study. Polystyrene tissue culture flasks (CellStar®, Greiner Bio-one, Monroe, NC, USA) were plated at a concentration of 0.267 x 10^6^ cells/cm^2^ and incubated at 37°C in 5% CO_2_. The culture media was completely replaced after 24 hours. Once MSC colonies had formed, the cells were lifted from the flasks using Accutase (StemPro®, Thermo Fisher®) and plated onto new flasks. Cells were then fed with MSC proliferation media comprised of Alpha modification of Eagle’s medium (AMEM, Gibco^TM^, Thermo Fisher®) with 10% FBS, 1% penicillin/streptomycin/amphotericin B and 2.5% 1M HEPES buffer.

Following passaging, cells were grown in culture flasks to 80% confluence. Cells from passages 2, 4, 6, and 8 were frozen at a concentration of 10^7^ cells/mL in freezing media (autologous equine serum and 10% dimethylsulfoxide (Molecular Probes^TM^, Eugene, OR, USA). Cryovials (2mL, Greiner Bio-one, Monroe, NC, USA) were cooled to -80°C using a slow-cooling container (Mr Frosty^™^, Thermo Fisher®) followed by storage in liquid nitrogen.

### Trilineage potential

MSCs from passage 4 of four horses from the Standardbred, Thoroughbred and blood donor groups were assessed for trilineage potential. Each horse’s cells were sampled in triplicate. The potential for adipogenic, osteogenic and chondrogenic differentiation was determined for the MSCs samples through cell expansion according to the manufacturer’s instructions. Briefly, MSCs were plated on chamber slides (Lab-Tek, Thermo Fisher®) at at 1 x 10^4^ cells/cm^2^ for the evaluation of adipogenesis, and at 5 x 10^3^ cells/cm^2^ for the determination of osteogenic potential. The chondrogenesis assay used 0.25 x 10^6^ cells that were centrifuged at 1000 x g for 5 minutes to form a cell pellet. After 24 hours of growth in proliferation media, MSCs were grown using specialized media (StemPro® Adipogenesis, Osteogenesis, and Chondrogenesis Differentiation Kits, Thermo Fisher®). The cells were grown in the differentiation media in monolayer for 14 days for adipogenic and osteogenic lineage assays. Cells were grown in pellet culture for 21 days for the chondrogenic lineage assay.

An additional set of cells was made by combining the Thoroughbred, Standardbred and blood donor cells in equal proportions. These cells were used as a control. A control sample was made for each lineage (adipogenic, osteogenic, and chondrogenic). These cells were cultured and treated in a similar manner as the trilineage groups except that only proliferation media was used (no induction media).

All cells were fixed in 4% formaldehyde at the end of the culture periods and stained as described for the respective differentiation protocols [34]. Adipogenic cells were stained with Oil Red O. Osteogenic cells were stained with Alizarin Red S. Chondrogenic pellets were embedded in paraffin and stained with Alcian Blue and counterstained with hematoxylin and eosin. Five randomly selected regions of each of the samples were assessed, providing 45 images to be used for evaluation of each of the Standardbred, Thoroughbred, and blood donor groups. The presence or absence of differentiation was evaluated using ImageJ software (National Institutes of Health, Bethesda, Maryland, USA). Adipogenesis was determined by percentage Oil Red O staining over total area of cell coverage. Osteogenesis was measured as percentage of alizarin red-positive area over total area. Chondrogenesis was measured as percentage of alcian blue-positive area over total area of cell coverage.

### Blood typing

Five mL of blood was collected in heparinized tubes (Heparin Tube, BD Vacutainer®, San Jose, CA, USA) for blood typing at the Equine Parentage and Animal Services Centre at Massey University. Blood was screened for Aa Ca and Qa antigens as horses that are used for blood donation (universal donors) should be negative for Aa, Ca, and Qa antigens [35, 36].

### Flow cytometry

The methods and the efficacy of the selected cell markers were first validated in a pilot study prior to use on the study population. All antibodies used in the main assay were first validated in the pilot study. Flow cytometry assays for CD 11a/18, CD 44, CD 90, and MHC class II antigens were validated using bone marrow-derived MSCs or leukocytes [16, 21, 23]. The specific antibodies used are included in the supporting information (S1 Table). Erythrocytes were added to exclude non-specific binding [23]. Erythrocytes autofluorescence did not cause these cells to appear positive for the fluorochromes as has been seen in other studies [37]. Samples from three horses were used for each antigen for validation assays. MHC class I molecules were not tested as they are consistently expressed at high levels in equine bone-marrow derived MSCs [9, 16]. Antibodies used were those previously reported and listed in the supporting information [21, 23]. All of the antibodies used were fluorescence conjugated for direct immunofluorescence. Those antibodies that were distributed without a conjugated fluorescing label were conjugated using an antibody labelling system (Mix-n-Stain^™^ Dye Antibody Labelling Kit, Biotium, Fremont, CA, USA; LYNX Rapid Antibody Conjugation Kit, Bio-Rad Laboratories, Hercules, CA, USA) (see supporting information). Antibody titration was performed to assure the optimal dilution was used. Antibody concentrations of 1:10, 1:50, 1:100, and 1:200 were compared using the stain index equation [38]. The dilution with the highest stain index was used. The most appropriate dilutions identified are listed in the supporting information, and these dilutions were used in subsequent assays.

For the validation study, aliquots of MSCs or leukocytes were suspended in phosphate-buffered saline (PBS) to obtain a concentration of 25 x 10^3^ cells/μL. A 40 μL aliquot (1 x 10^6^ cells) was used for each flow cytometry assay. The cells were incubated with a viability stain (1ul/500ul cells, Efluor 780^™^, eBioscience^™^, San Diego, CA, USA) for 30 minutes on ice and protected from visible light. The cells were then washed with PBS and the diluted antibodies for CD11a/18, CD44, CD90, and MHC class II molecules added were added at the same time. The mixture was incubated on ice and protected from visible light for 30 minutes. The samples were then washed with 2mL PBS to remove excess (non-bound) antibody and fixed in 3% paraformaldehyde for 20 minutes. After a final wash and dilution in 1mL PBS, the cells were evaluated on a flow cytometer (BD FACSVerse^TM^, San Jose, CA, USA). Data were collected on 1 x 10^4^ large cell events (small debris was ungated) for each sample.

All data were compensated and corrected for autofluorescence using cytometric capture beads (BD^™^ CompBeads, San Jose, CA, USA), single stains, and all-fluorochromes-minus-one compensation tubes. Compensation for any spectral overlap between fluorochromes and data evaluation was performed using specialized flow cytometry software (FlowJo®, Ashland, OR, USA).

Gating was performed on a hierarchy format with, first, cells being isolated over a time frame that provided consistent cell acquisition data. Then viable cells were selected according to their low viability stain uptake. A mononuclear cell subset was selected by graphing on forward cell scatter area and height. Finally a large cell population was selected. This gated cell population was used to determine cell marker expression.

After initial gating to identify an appropriate cell population for further analysis, these cells were gated to identify populations of cells positive and negative for each of the markers. The populations were gated using both unstained cells and stained cells known to be negative or positive for the marker. Data were reported as the percent of cells in this population that showed fluorescence for a specific marker. Both LK and JR (acknowledgments) performed independent data analysis prior to finalizing the results.

After antibody validation, a sample of 1 x 10^6^ equine MSCs in the fourth passage was used to compare expression levels from MSCs immediately removed from culture and those that had been cryopreserved 24 hours prior. Samples from three horses were used in this part of the study. Expression of the cell markers were compared using a Chi-Square test for proportional populations. This pilot study was performed to confirm that cryopreserved cells could be used to accurately depict the cell marker expression.

After these validation steps were performed, MSCs derived from bone marrow samples of the 36 test horses were examined. Cell surface expression of CD11a/18, CD44, CD90, and MCH class II molecules at culture passages 2, 4, 6 and 8 were analysed for each of these horses. These passages were selected to give an overview of marker expression during the early culture period, when MSCs are commonly utilized for therapy because they are more proliferative and therefore provide sufficient numbers for treatment, and have a greater potential for differentiation than later stage passages [39].

### Data analysis

Flow cytometry and trilineage data were not normally distributed, and followed a beta distribution. Data transformation did not produce normally distributed data. Summary statistics for cell marker expression are expressed as median (interquartile range [IQR]). Data points were classified as outliers if they were greater than 1.5 times the IQR below the 25^th^ quartile or greater than 1.5 times the IQR above the 75^th^ quartile. Data for each molecular marker were plotted and each variable had a beta distribution. Beta regression was performed to identify breed (Standardbred; Thoroughbred), blood donor status (universal donor; non-donor) and temporal effects (passage 2, 4, 6 and 8) on cell marker expression of the gated cell population using statistical software (Betareg package in R, Version 3.4.3, R Core Development Team) [40]. Goodness of fit of the model was determined with a likelihood ratio test, with significance at p < 0.05. Post-hoc analyses by Wilcoxon rank sum and Mann-Whitney U tests were performed to identify the source of significant differences (if identified) among passages within breed, and between breeds at each passage. Similarly, Wilcoxon rank sum tests were used to then identify significant differences among passages within the universal donor and non-donor horse groups, and between universal donor and non-donor horses at each passage. The latter comparisons were restricted to Standardbreds, as there were no Thoroughbred universal donors. All differences were considered significant at p<0.05. Chi-Square statistics were calculated for cryopreservation assays to determine if there was a difference in marker expression between fresh and cryopreserved cells. Differences and correlations were considered significant at p<0.05.

## Results

### Standardbred, Thoroughbred and blood donor MSCs show appropriate trilineage differentiation

**Fig 1:**
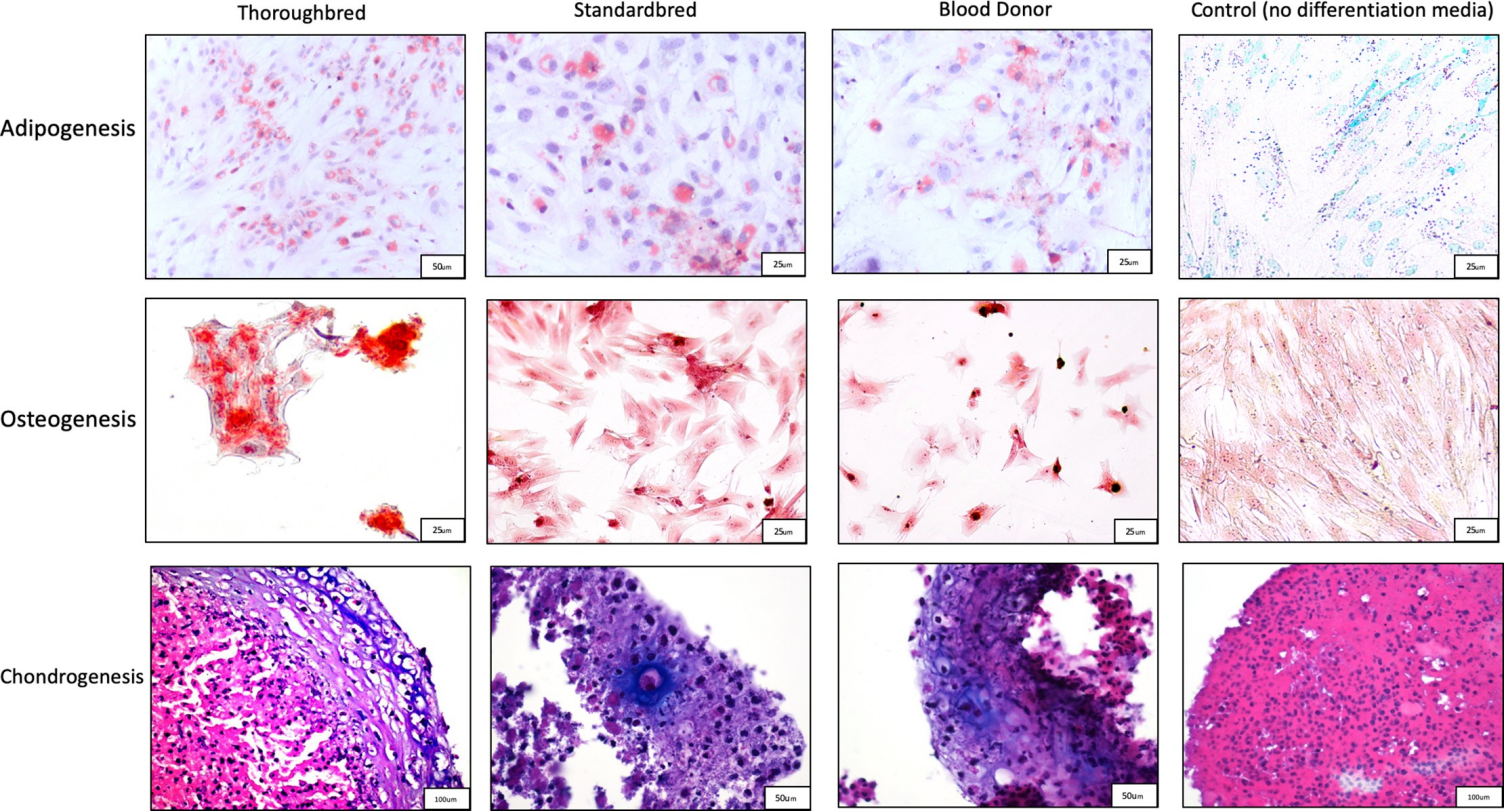
Trilineage differentiation is seen in all groups of MSC treated with induction media. Trilineage differentiation assays were performed on Standardbred, Thoroughbred, and blood-donor MSCs. Cells placed in induction media showed differentiation down adipogenic, osteogenic, and chondrogenic lines. Control MSCs cultured in media without induction agents showed no differentiation.

Four Standardbred, Thoroughbred, and blood donor MSC samples from passage 4 were tested in triplicate for differentiation towards adipogenic, osteogenic and chondrogenic lineages (Fig 1). These groups were compared to MSCs treated with MSC proliferation media only (no differentiation media). Lipid deposits could be seen in the adipogenic induction plates and lipid staining was significantly greater than control (non-induced) MSCs for the Standardbred (p = 0.010), Thoroughbred (p = 0.00039) and blood donor (p = 0.020) groups. Calcium deposits were present in the osteogenic induction plates and staining was significantly greater than control (non-induced) MSCs for the Standardbred (p = 0.00016), Thoroughbred (p = 0.000076) and blood donor (p = 0.000057) groups. Glycosaminoglycan staining was seen in the chondrogenic induction pellets and staining was significantly greater than control (non-induced) MSCs for the Standardbred (p<0.00001), Thoroughbred (p<0.00001) and blood donor (p = 0.00027) groups.

### MSC inclusion and exclusion antibodies were validated and cryopreservation did not alter marker expression

A full description of the antibody validation and dilutions are included in the supporting information.

Cryopreserved and fresh MSCs at the fourth passage were compared for their cell marker expression levels to assure that cryopreserved cells would appropriately represent fresh cell expression. There was no significant difference in surface marker expression on fresh samples as compared to cryopreserved samples (Chi-Square values 0.133–0.602) for CD11a/18 (p = 0.44), CD44 (p = 0.64), CD90 (p = 0.53) and MHC class II (p = 0.71). Cryopreserved cells were used for subsequent assays.

**Fig 2:**
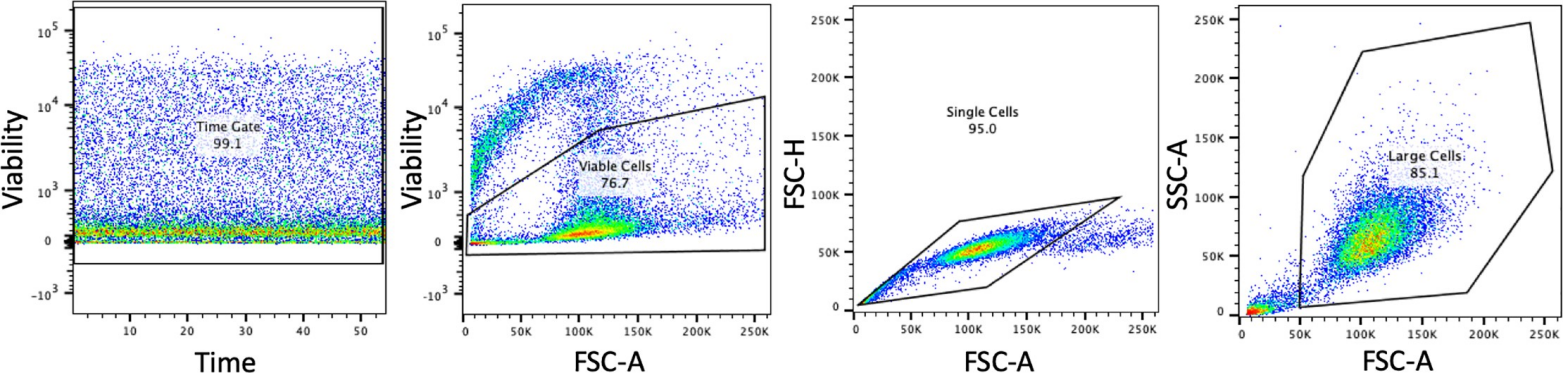
Gating scheme for MSC selection used in flow cytometry. Representative dot plots show the gating scheme that was used prior to quantification of MSCs positive and negative for the desired marker.

**Fig 3:**
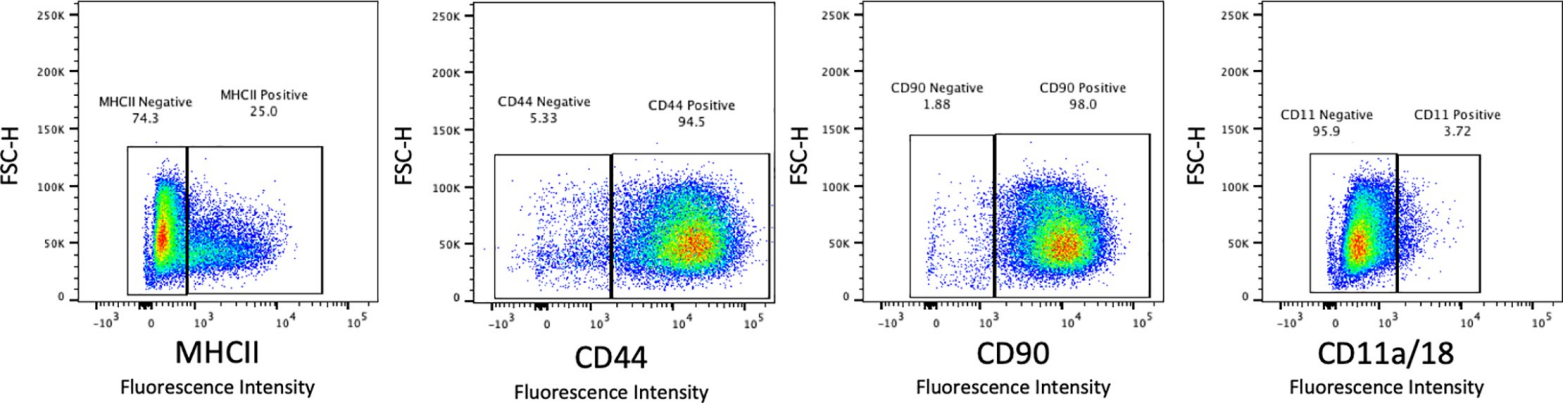
Positive and negative cell populations for each antibody illustrate marker expression in MSCs. A representative MSC sample from passage 2 shows MHC class II, CD44, CD90 and CD11a/18 expression.

The gating scheme used for flow cytometric evaluation of a final large, viable cell population is shown in Fig 2. The antibodies used in this study showed appropriate binding to PBMCs or MSCs and did not bind to erythrocytes (Fig 3). Positively- and negatively-gated populations for each antibody are shown in Fig 3.

### Blood typing reveals blood donor and non-blood donor type horses

All Standardbreds and Thoroughbred horses were blood-typed to identify the presence of Aa, Ca and Qa antigens on their erythrocytes. All Thoroughbred horses were positive for at least one of the erythrocyte antigens. Eight of the 18 Standardbreds were negative for all three antigens. These 8 horses were categorized as universal blood donor horses for comparison of universal blood donor and non-blood donor groups.

### Beta regression models to understand multiple variables

In the multivariable model for MHC II expression, passage number (p<0.001) and breed (p<0.001) but not donor status (p = 0.70) were significant contributors to variance; the model was significant (df = 5; Chisq = 30.67; p<0.001). In the multivariable model for CD 11a/18 expression, breed (p = 0.003) and blood donor status (p = 0.04) but not passage number (p = 0.11) were significant contributors to variance; the model was significant (df = 5; Chisq = 13.32; p = 0.004). In the multivariable model for CD 44 expression, breed (p = 0.005) and blood donor status (p = 0.04) but not passage number (p = 0.19) were significant contributors to variance; the model was significant (df = 5; Chisq = 10.60; p = 0.014). In the multivariable model for CD 90 expression, breed (p<0.001), blood donor status (p = 0.001) and passage number (p = 0.013) were significant contributors to variance; the model was significant (df = 5; Chisq = 26.11; p<0.001).

### Analysis of marker expression by breed shows significant differences between Standardbred and Thoroughbred MSCs

**Fig 4:**
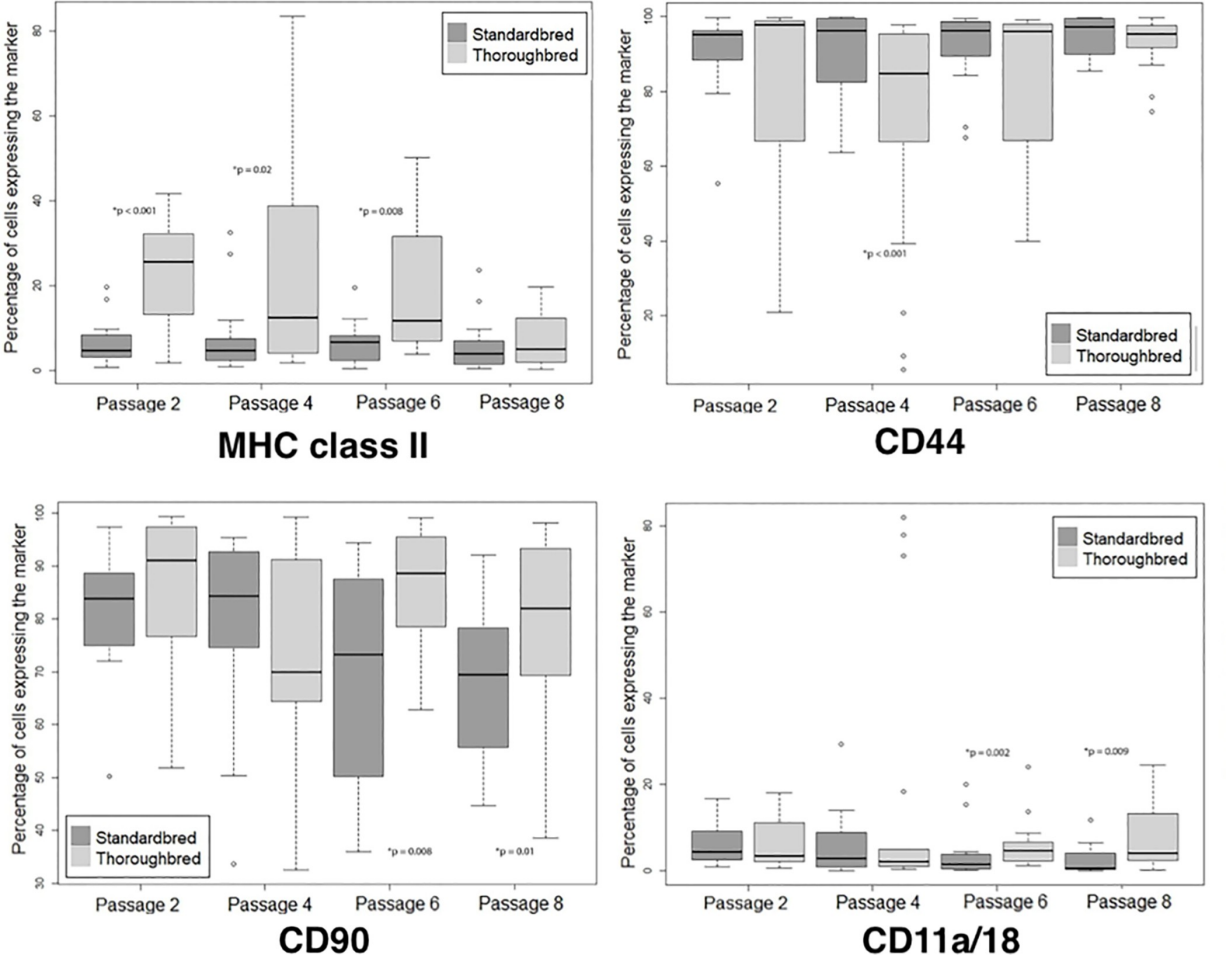
Marker expression by breed. These graphs show the breed differences in marker expression over the time points (passages). Median marker expression is represented in each of the graphs as a percent of cells that show the marker as compared to the total gated cell population. The IQR is shown as the top and bottom of the box. Extreme values are shown with the error bar. Excluded data points are listed with a bullet. Passages with significantly different expression between the Thoroughbred and Standardbred populations are indicated by an asterisk.

When marker expression was compared between the breeds, several markers showed significant differences (Fig 4). Standardbreds were significantly lower in their expression of MHC class II overall (p<0.001) and in particular during the early and middle passages as compared to Thoroughbreds (p<0.001 at passage 2; p = 0.02 at passage 4, p = 0.008 at passage 6) (Fig 4). Expression levels were similar at passage 8 only. Overall, MHC class II expression was low for both phenotypes, though Thoroughbreds showed higher variation and were more likely to be high at early passages (Fig 4).

CD11a/18 expression was also low through all passages with the median not exceeding 5% at any passage number (Fig 4). CD11a/18 expression was significantly higher in Thoroughbreds over all time points (p = 0.001), especially at the later passages (p = 0.002 at passage 6; p = 0.009 at passage 8) (Fig 4). CD44 expression was high through all passages with the mean > 80% in both groups. Its expression was significantly higher in the Standardbred population over all time points (p = 0.002) and most impressively at passage 4 as compared to Thoroughbreds (p<0.001). CD90 expression was also high through all passages with > 70% of MSCs expressing CD90 in both groups. CD90 was expressed significantly more often in Thoroughbred MSCs over all time points (p<0.001) and, in particular, at passages 6 (p = 0.008) and 8 (p = 0.01) as compared to Standardbred MSCs.

Comparing changes in expression with passage, within the Thoroughbred group MHCII expression differed significantly between passages 2 (p<0.001), 4 (p = 0.007), 6 (p = 0.005) and passage 8. It did not significantly differ among passages within the Standardbred group. CD11a/18 expression did not significantly differ among passages for the Thoroughbred group. CD11a/18 expression in Standardbreds differed significantly between passages 2 (p = 0.01), 4 (p = 0.013) and passage 8, and between passages 4 and 6 (p = 0.047). There were no significant differences in CD44 expression among passages within the Thoroughbred or Standardbred groups. CD90 expression within the Thoroughbred group differed significantly between passages 2 and 4 (p = 0.034), and between passages 4 and 6 (p = 0.01). CD90 expression within the Standardbred group differed significantly between passages 2 and 8 (p = 0.002), and between passages 4 and 8 (p = 0.002).

### Analysis of marker expression by blood type shows significant difference between universal blood donor non-blood donor MSCs

**Fig 5:**
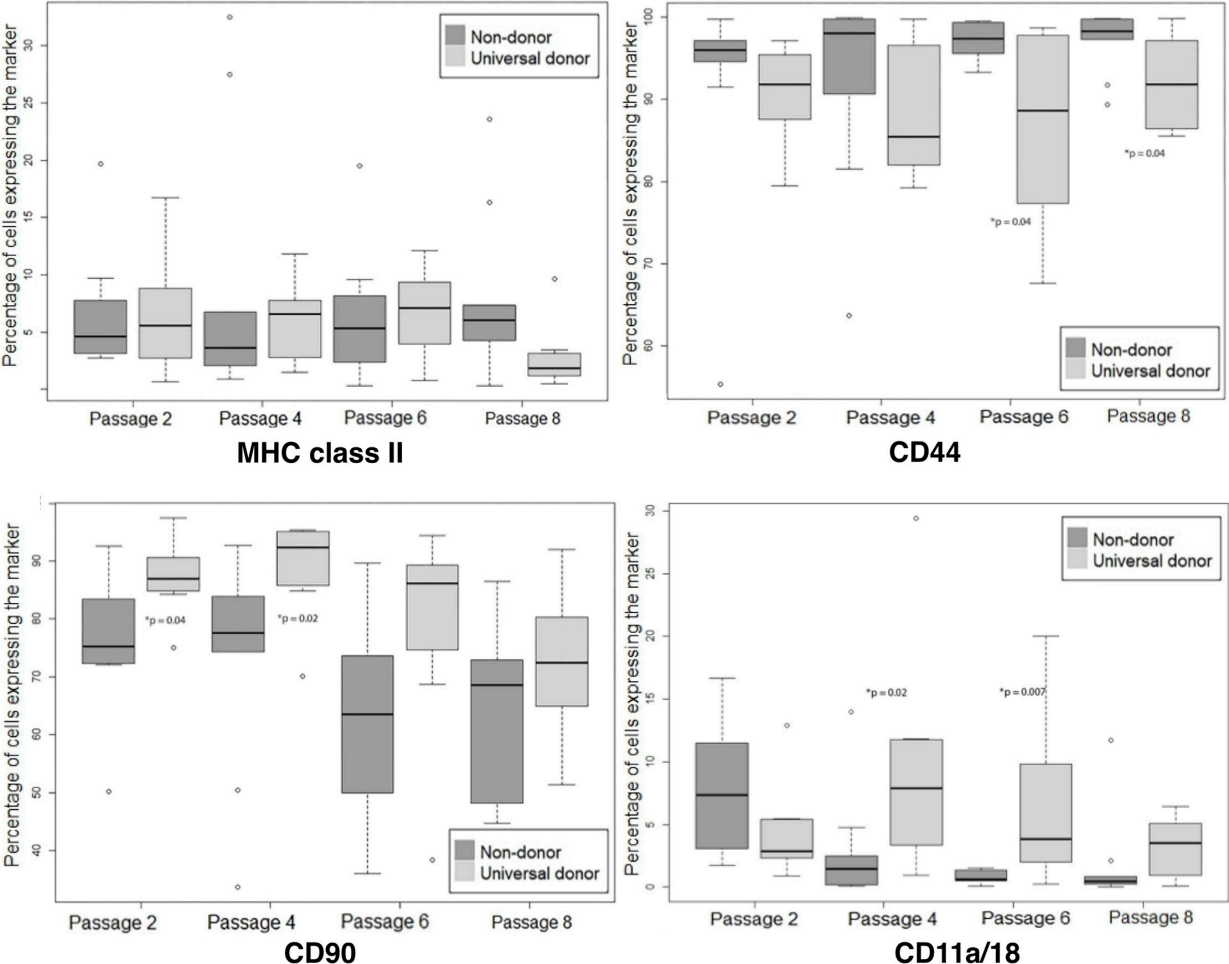
Marker expression by blood donor status. These graphs show the differences in marker expression between blood donor horses and non-blood donor horses over successive passages. Median marker expression is represented in each of the graphs as a percent of cells that show the marker as compared to the total gated cell population. The IQR is shown as the top and bottom of the box. Extreme values are shown with the error bar. Excluded data points are listed with a bullet. Passages with significantly different expression between the Thoroughbred and Standardbred populations are indicated by an asterisk.

When the 8 universal blood donor horses (all were Standardbreds) were compared to the 10 non-blood donor Standardbred horses, there were significant differences in MSC expression of CD11a/18, CD44, and CD90 molecules (Fig 5). Expression of MHC class II was not significantly different between the non-blood donor horses as compared to the universal blood donor horses (p = 0.72). Expression of CD11a/18 was lower in the non-blood donor horses at passages 4 (p = 0.020) and 6 (p = 0.007). CD44 expression was consistently high with a median of > 80% for both groups. Non-blood donor horses had significantly higher CD44 expression compared to blood universal donor horses at passages 6 (p = 0.040) and 8 (0.040). CD90 expression was significantly higher in universal blood donor MSCs at passages 2 (p = 0.040) and 4 (p = 0.020).

### Correlation among cell markers shows MHC class II, CD90 and CD11a/18 are expressed similarly

When all groups of horses and all passages were analysed, cell markers showed correlation in their expression with the other measured markers (Table 1) CD11a/18 expression was positively correlated with CD90 and MHC class II (p<0.001, Table 1). Expression of CD90 and MHC class II were positively correlated (p<0.001, Table 1). CD44 expression was not correlated with that of any other cell marker.

**Table 1:** Correlation of marker expression.

	CD11a/18	CD44	CD90	MHC class II
CD11a/18	1	0.0647 (0.483)	**0.547** **(< .0001)**	**0.655** **(< .0001)**
CD44	0.0647 (0.483)	1	-0.0571 (0.535)	-0.0741 (0.421)
CD90	**0.547** **(< .0001)**	-0.0571 (0.535)	1	**0.393** **(< .0001)**
MHC class II	**0.655** **(< .0001)**	-0.0741 (0.421)	**0.393** **(< .0001)**	1

Marker expression correlation is listed for all data through all time points. The degree of correlation (R value) is listed followed by the p-value (in parentheses). Bold values show significant correlations.

## Discussion

There are several breed and blood donor-status effects on MSC marker expression, influenced by the passage number. Bone marrow-derived MSCs from Standardbreds showed significantly less MHC class II expression at early passages as compared to Thoroughbreds. Evidence of breed associated differences in cell surface expression may explain, in part, why such large differences in the literature exist for MHC class II expression by equine MSCs. Paebst *et al*. *(*2014) showed the mean percent of MSCs from Warmblood horses expressing MHC class II to be 0.25% at passage 3 [20]. Schnabel *et al*. (2014) reported that the mean percent of MSCs from Thoroughbred horses expressing MHC class II at passage 2 was 59.0% ± 26.3 and at passage 4 was 46.8% ± 36.2 [9]. In comparison with the expression data from Schnabel *et al*. (2014), our Thoroughbred horse data showed a decreased median MHC class II expression at 18.5% and 12.5% for passages 2 and 4, respectively. It is possible that this difference in Thoroughbred expression between studies is due to breed variation secondary to gene flow as New Zealand based Thoroughbreds were used for the current study [41, 42].

The effect of blood donor status on MSC phenotype has not previously been studied in horses. It appears that the lack of immunogenic antigens on the surface of the erythrocyte (blood donor-status) does not correlate with a lack of MHC class II on the MSC surface as there was no significant difference in MHC class II expression between blood donors and non-blood donor horses. This observation was limited to Standardbreds, as MSC samples from universal donor-type Thoroughbreds were not identified during the screening process for recruitment to the study. The use of other blood donors of other breeds would have assisted our analysis.

One finding in the current study and in those previously published is that some horses with high initial MHC class II expression show a decreased expression over time [9]. Five of 11 highly expressing samples in Schnabel *et al*. 2014 decreased to less than 2% of cells expressing MHC class II [9]. Six of 14 horses in our study with higher MHC class II expression at passage 2 decreased to less than 5% by passage 8. While decreased expression may be beneficial insofar as these cells are less likely to stimulate immune responses in the recipient than MHC class II high cells, MSCs at these late passages have deficits as compared to their younger relatives [43]. More highly passaged MSCs show an altered phenotype (decreased expression of MSC markers), have decreased proliferation rates, and develop an altered morphology [39, 43, 44]. For these reasons, older MSCs may be considered less desirable for treatment of disease.

MSCs were consistently positive for CD44 in this study, and this marker was highly expressed in all of the MSC populations examined. This consistent high expression in MSCs is in agreement with previously published studies [9, 16, 21, 22].

In the current study, the percent of MSCs positive for CD90 was high through all passages. Ranera *et al*. (2011) found 90% positive expression of CD90 at passage 3 in equine bone marrow-derived MSC sample, which is comparable to the results attained in our study [45]. Universal blood donor-type Standardbreds had significantly higher CD90 expression than non-blood donor Standardbreds over all time points (p<0.001). CD90 is known to be involved in cell proliferation, survival, migration and regulating differentiation [46, 47]. When CD90 gene expression is suppressed using interfering RNA, cells move towards differentiation [47]. A high level of CD90 expression in the MSC population appears important for maintaining pluripotency [46–48]. Therefore, the universal blood donor-type Standardbred may provide superior MSCs than the non-blood donor Standardbreds.

CD11a/18, an adhesion protein used by leukocytes to adhere to endothelium, was used in this assay to identify contaminating cells [25, 49]. CD11a/18 expression was significantly lower in Standardbreds as compared to Thoroughbreds and in non-blood donor horses as compared to universal blood donors. The cause of increased leukocyte contamination in some groups is unclear as the MSC isolation regimes were identical. The evidence of higher leukocyte contamination may be related to a difference in the number and ratio of myeloid cells in the bone marrow in one breed as compared to the other, though no studies have been performed to corroborate this hypothesis. Most importantly, CD11a/18 expression was low in all groups (Figs 4 and 5).

Expression of each marker was compared to one another to determine if there were significant correlations of expression (Table 1). Most interestingly, there was no correlation between CD44 and CD90 (r = -0.0571, p = 0.535). Both of these antigens are commonly found on cultured bone marrow-derived equine MSCs [9, 23]. Although these markers were both consistently highly expressed on our MSCs, based on findings in the current study, their cell functions do not appear to be linked. Their expression has seldom been linked in previous MSC marker expression studies [47]. The correlation of expression in CD11a/18, CD90 and MHC class II may due to their expression on a small number of contaminating non-MSCs that represented by the total CD11a/18 positive cell population.

Studies define a cell population expressing a marker ≥90% of the time as “positive” for the marker while a cell population expressing a marker ≤10% of the time are “negative” for the marker [50]. Overall, our MSCs are CD11a/18 negative, CD44 and CD 90 positive, and MHC class II heterogenous.

In conclusion, universal blood donor-type Standardbred horses appear less likely to cause an MHC class II driven immune reaction and have high levels of bone marrow-derived MSC markers. As bone marrow-derived MSCs express MHC class I, further testing will be needed to determine whether these early passage universal blood donor-type Standardbred MSCs can be used in an allogenic manner or if haplotyping will be necessary.

## Supporting information

S1 Table Antibodies listed showed high fluorescence in the appropriate positive cell population and negative to poor fluorescence on the negative cell population. The optimal dilution according to the stain index is listed.
(DOCX)

## Abbreviations

CD: Cluster of differentiation
FBS: fetal bovine serum
MHC: major histocompatibility complex
MSC: mesenchymal stem cell
PBS: phosphate-buffered saline
