# Supplementary material for: Blood type and breed-associated differences in cell marker expression on equine bone marrow-derived mesenchymal stem cells including major histocompatibility complex class II antigen expression
Source: PLoS One. 2019 Nov 20;14(11):e0225161. doi: 10.1371/journal.pone.0225161 (PMC6867698; doi:10.1371/journal.pone.0225161)
Supplement: S1 Table — The optimal dilution according to the stain index is listed. (DOCX) [file pone.0225161.s001.docx]

**Supporting Information**

| **Antibody Clone** | **Distributer, Catalog number** | **Conjugated fluorochrome or Kit used for labeling antibody** | **Host Species** | **Target Species** | **Ig Type** | **Dilution** | **Positive equine cell populations (publication reported)** | **Negative equine cell populations (publication reported)** |
| --- | --- | --- | --- | --- | --- | --- | --- | --- |
| CD 11a/18 CVS9 | Bio-Rad, MCA1081PE | PE | Mouse | Equine | IgG1 | 1:50 | Leukocytes [21] | Erythrocytes, MSCs [23] |
| CD44 CVS18 | Bio-Rad, MCA1082GA | Biotium Mix-N-Stain CF647 Antibody Labelling Kit | Mouse | Equine | IgG1 | 1:200 | Leukocytes, MSCs [21, 23] | Erythrocytes [23] |
| CD90 Thy-1 | Washington State University Monoclonal Antibody Center, DH24A | LYNX Rapid PerCP-Cy5.5 Antibody Conjugation Kit | Mouse | Equine | IgM | 1:100 | Granulocytes, MSCs [21, 23] | Lymphocytes [23] |
| MHC class II CVS20 | Bio-Rad, MCA1085F | FITC | Mouse | Equine | IgG1 | 1:100 | Lymphocytes [21] | Granulocytes [23] |

**Table 1. Antibodies used for flow cytometry assays.** Antibodies showed high fluorescence in the appropriate positive cell population and negative to poor fluorescence on the negative cell population. The optimal dilution according to the stain index is listed.
